# Functional Properties of High-Pressure Assisted Enzymatic Tamarind Kernel Protein Hydrolysate and Foam-Mat Powder Characteristics as Affected by HPMC Concentration and Drying Temperature

**DOI:** 10.3390/foods15030511

**Published:** 2026-02-02

**Authors:** Warangkana Sompongse, Thanavuth Vutthidech, Worawan Hongviangjan

**Affiliations:** Department of Food Science and Technology, Faculty of Science and Technology, Thammasat University, Khlong Luang, Pathumthani 12120, Thailand

**Keywords:** foam-mat drying, high-pressure processing, protein hydrolysate, tamarind kernel powder

## Abstract

The functional properties of high-pressure processing (HPP)-assisted protein hydrolysate from tamarind kernel powder (TKP-HD) and the physicochemical characteristics of its foam-mat powder were studied. TKP-HD consisted of more non-polar than polar amino acids, with higher solubility at pH 5 and 7 than soy protein isolate (SPI) but lower than egg white (EW). The water-binding capacity of TKP-HD increased at pH 5 while TKP-HD had a higher foaming capacity than SPI at pH 5, and the highest oil-binding capacity. The physicochemical properties of TKP-HD after foam-mat drying were investigated using 1 and 1.5% (*w*/*w*) hydroxypropyl methylcellulose (HPMC), with drying at 60, 70, and 80 °C. Samples with 1.5% HPMC had lower water activity than those with 1% HPMC at all drying temperatures. The sample with 1% HPMC had higher antioxidant capacity at 60 °C than at 70 °C, but this decreased at 1.5% HPMC. Samples with 1.5% HPMC and dried at 60 °C recorded the highest solubility and viscosity, with increased porosity of the powder structure. The most suitable foam-mat drying conditions for TKP-HD were the addition of 1.5% HPMC and drying at 60 °C.

## 1. Introduction

Tamarind kernel powder (TKP) is widely perceived as a low-value by-product of tamarind production, contrasting with its high nutritional potential. TKP is rich in antioxidants, proteins (15–21%), essential amino acids, fats (3–8%), carbohydrates (50–60%), and minerals [1]. TKP contains higher levels of essential amino acids, including isoleucine, leucine, lysine, methionine, and cysteine, than widely consumed legumes such as groundnuts, cowpeas, chickpeas, and soybeans [2,3], indicating its potential for use as a value-added ingredient in food applications. However, the protein matrix of TKP is associated with polysaccharide granule surfaces via hydrogen bonds, leading to the formation of polysaccharide–protein complexes [4,5], and identifying effective approaches to separate TKP proteins from polysaccharides remains a critical prerequisite for their functional applications. Several reports have investigated methods for separating TKP proteins, ranging from conventional approaches, including alkaline extraction [6], multi-solvent extraction [7], and isoelectric precipitation [8], to novel techniques such as ultrasound-assisted processing [9].

Enzymatic hydrolysis involves the breakdown of proteins through the action of proteolytic enzymes under optimal temperature and pH conditions, resulting in peptides of varying sizes and free amino acids [10]. Several studies have reported that enzymatic hydrolysis enhances functional properties, including solubility, emulsifying, foaming, and gelling characteristics, while concurrently modifying protein–protein and protein–polysaccharide interactions [7,11,12]. Therefore, this process is an effective approach for disrupting and separating TKP proteins from complex polysaccharide–protein structures. Papain, a cysteine protease derived from papaya latex, is extensively applied in the food industry to produce protein hydrolysates that function as flavor enhancers, food supplements, and functional food ingredients [13]. Previous studies reported that papain-mediated hydrolysis of tamarind proteins yielded hydrolysates with the highest antioxidant activity among tested enzymes, alongside lower processing costs [7].

High-pressure processing (HPP) is a non-thermal technology that has been commercially implemented for decades in numerous countries to achieve pasteurization and extend shelf life. At the molecular level, HPP can dissociate noncovalent interactions, including hydrophobic, electrostatic (ionic), and hydrogen bonds [14,15]. HPP can also modify protein conformation and structure. Tertiary and quaternary protein structures are most sensitive to pressure, as they are stabilized by hydrophobic and electrostatic interactions that are pressure sensitive. Secondary structural changes typically occur only at higher pressures, whereas the primary structure, stabilized by covalent bonds, is preserved [16,17,18]. HPP has been proposed as an effective approach to enhance enzymatic hydrolysis by increasing protein unfolding and exposing additional cleavage sites to proteolytic enzymes [19]. Several studies on HPP-assisted enzymatic hydrolysis demonstrated that the application of HPP, in combination with trypsin for flaxseed proteins [20] and with alcalase for quinoa, lentil, and Great Northern bean proteins [21,22,23], resulted in increased degrees of hydrolysis and improved functional properties of the resulting hydrolysates.

High-pressure processing (HPP) has been shown to induce structural modifications in plant proteins through the disruption and reorganization of non-covalent interactions. Sricheevachart et al. [3] reported that TKP treated by HPP exhibited alterations in protein secondary structure, as evidenced by changes in the amide I and II regions, along with enhanced release of bioactive compounds. Such pressure-induced conformational rearrangements can promote partial protein unfolding and the exposure of enzyme-accessible cleavage sites. These structural changes are expected to influence peptide size and surface hydrophobicity following enzymatic hydrolysis, thereby affecting key functional properties such as solubility, oil-holding capacity, and foaming behavior. Accordingly, combining HPP with enzymatic hydrolysis provides a rational strategy for enhancing the functional performance of protein hydrolysates.

Foam-mat drying is a cost-effective and energy-efficient approach for the preservation of heat-sensitive foods such as fruits, vegetables, dairy products, and protein-rich matrices [24,25]. The process involves drying liquid or semi-solid foods after mixing them with stabilizing and/or foaming agents (hydroxypropyl methylcellulose; HPMC, carboxymethyl cellulose; CMC, maltodextrin, egg white, and plant proteins) to form a stable foam, which is subsequently subjected to hot air drying at 50–80 °C [26]. Drying is performed at relatively low temperatures to form a thin, porous honeycomb sheet or mat. The incorporation of fine air bubbles increases the effective surface area, promoting rapid moisture evaporation [25,26,27]. This results in more rapid drying kinetics and also ensures superior retention of color, flavor, and nutritional quality compared to conventional hot air drying [26]. The high porosity of the resulting powder contributes to improved functional properties, particularly enhanced solubility and dispersibility during reconstitution [28]. Previous research found that foam-mat drying promoted the functional properties and bioactivity of protein hydrolysates, as evidenced by improved emulsion and foam stability in squid head protein hydrolysates [29], and enhanced the preservation of phenolic compounds contributing to antioxidant activity in rice bean protein hydrolysates [30].

HPP-assisted enzymatic hydrolysis of plant proteins has been extensively reported. Our previous study [4] focused on the effects of high-pressure pretreatment and hydrolysis time on the degree of hydrolysis and antioxidant properties of tamarind kernel protein hydrolysates. Interestingly, the present study is the first to investigate the functional properties of protein hydrolysates from tamarind kernel powder (TKP-HD) produced via HPP-assisted enzymatic hydrolysis, as well as the physicochemical characteristics of dried foam-mat TKP-HD. The functional properties of TKP-HD, including color, chemical composition, amino acid profile, solubility, water-binding capacity, oil-holding capacity, as well as foaming capacity and stability, were compared with egg white (EW) and soy protein isolate (SPI). The physicochemical characteristics of foam-mat-dried TKP-HD were examined in terms of chemical composition and water activity, with the antioxidant activity assessed using the DPPH free radical scavenging assay and the ferric reducing antioxidant power (FRAP) assay. Functional properties such as solubility, water-binding capacity, viscosity, and powder microstructure were characterized using scanning electron microscopy (SEM).

## 2. Materials and Methods

### 2.1. Materials and Chemicals

Tamarind kernel powder (TKP) with a moisture content of 6.53% and a protein content of 19.52% [4] was purchased from Freshy Thai Co., Ltd. (Chachoengsao, Thailand). Papain (from papaya latex), 2,2-diphenyl-1-picrylhydrazyl (DPPH), and 2,4,6-tripyridyl-s-triazine (TPTZ) were purchased from Sigma-Aldrich (St. Louis, MO, USA). Egg white (EW) and soy protein isolate (SPI) were purchased from Thai Food and Chemical Co., Ltd. (Samut Prakan, Thailand) and Food EQ Co., Ltd. (Bangkok, Thailand), respectively. Hydroxypropyl methylcellulose (HPMC) was purchased from Krungthep Chemical Co., Ltd. (Bangkok, Thailand). All other chemicals used in this study were of analytical grade.

### 2.2. High Hydrostatic Pressure Processing of Tamarind Kernel Powder

High-pressure processing of tamarind kernel powder (HPP-TKP) was performed following a previously reported method [4]. Briefly, a TKP suspension in deionized water (10% *w*/*w*) was homogenized at 5000 rpm for 5 min using a homogenizer (T18 Digital ULTRA-TURRAX^®^, IKA Werke GmbH & Co. KG, Staufen, Germany). The homogenized mixture was sealed in polyethylene bags and subjected to high hydrostatic pressure treatment at 400 MPa for 30 min in a water-filled pressure chamber (HPP 600 MPa, BaoTou KeFa High Pressure Technology, Baotou, China). The samples were then lyophilized and stored at −18 °C before further protein extraction.

### 2.3. HPP-TKP Protein Extraction

The extraction of proteins from HPP-TKP followed the procedure reported by [4]. Defatted HPP-TKP (5 g) was initially treated with 95% ethanol (5 mL) to prevent agglomeration and dispersed in distilled water (245 mL). After stirring for 30 min, the slurry was heated at 90 °C for 40 min in a water bath (WNB Series, Memmert GmbH & Co. KG, Schwabach, Germany) with continuously stirring, stored for 24 h, and centrifuged (5000 rpm, 20 min), Finally, the precipitate was rinsed with ethanol, freeze-dried, and stored at −18 °C until subsequent hydrolysis.

### 2.4. HPP-TKP Protein Hydrolysis

The HPP-TKP protein hydrolysis was conducted according to the method described by [4]. HPP-TKP protein (1 g) was dispersed in phosphate-buffered saline (40 mL, pH 7) and incubated at 60 °C for 30 min in a shaking incubator (JSSI-100C, JS Research Inc., Gongju, Korea). Enzymatic hydrolysis using papain (enzyme-to-substrate ratio, 1:10 *w*/*w*) was carried out at 60 °C for 4 h (Degree of hydrolysis = 23.49%). To terminate the proteolysis, the slurry was heated at 90 °C for 10 min and subsequently lyophilized. The obtained HPP-TKP protein hydrolysis (TKP-HD) was stored at −18 °C before analysis.

### 2.5. Determination of Functional Properties of TKP-HD

The functional properties of TKP-HD were compared, on an ingredient-level basis, with egg white (EW) and soy protein isolate (SPI), which are widely used reference proteins representing animal- and plant-derived protein sources. EW and SPI were used as upper-bound functional benchmarks for ingredient-level evaluation of TKP-HD.

#### 2.5.1. Color

The color parameters of the samples were measured using a colorimeter (ColorFlex CX2687, HunterLab, Reston, VA, USA) and reported according to the CIELAB color system, expressed as L* (lightness), a* (redness/greenness), and b* (yellowness/blueness).

#### 2.5.2. Chemical Composition

The samples were analyzed for moisture, protein, and fat content following the AOAC procedures [31].

#### 2.5.3. Amino Acid Profiles

The amino acid profiles of TKP and TKP-HD were determined according to the method described by [32]. Briefly, samples (0.008 g) were hydrolyzed with 6 N HCl for 1 h, and the amino acid compositions were analyzed using an amino acid analyzer (L-8500, Hitachi High-Tech, Tokyo, Japan).

#### 2.5.4. Solubility and Water-Binding Capacity

A 0.2 g sample was dissolved in 20 g of distilled water and adjusted to pH 5 or 7 using 0.1 N NaOH. The mixtures were vortex-mixed for 1 min and subsequently centrifuged at 4000× *g* for 15 min. The supernatant was gently poured into an aluminum can, and the residual liquid was removed from the sediment as completely as possible. The wet precipitate was weighed, and both the supernatant and precipitate were dried in a hot air oven at 105 °C overnight. The solubility was calculated according to the equation presented below [33,34], with the water-binding capacity expressed as grams of water bound per gram of sample.

(1) $$Solubility\left(\%\right)=\frac{mass\;of\;dried\;supernatant}{mass\;of\;sample}\times 100$$

#### 2.5.5. Oil-Holding Capacity

The samples (0.5 g) were vortex-mixed with soybean oil (20 mL) for 30 s and equilibrated for 1 h at room temperature. The mixtures were then centrifuged at 4000× *g* for 30 min at 25 °C. The supernatant was decanted and weighed, with the oil-holding capacity expressed as grams of oil bound per gram of sample [34].

#### 2.5.6. Foam Capacity and Foam Stability

A 0.2 g sample was dispersed in 20 mL of distilled water, and the pH was adjusted to 5 or 7 with 0.1 N NaOH. The mixture was then homogenized at 15,000 rpm for 2 min using a homogenizer (T18 Digital, IKA Werke GmbH & Co. KG, Staufen, Germany). The foam capacity and stability were evaluated by measuring the foam volume before and immediately after homogenization and after 30 min of standing, respectively. Foam capacity and foam stability were calculated using the equations presented below [34].

(2) $$Foam\;capacity\left(\%\right)=\frac{\left(volume\;after\;homogenization-volume\;before\;homogenization\right)}{volume\;before\;homogenization}\times 100$$

(3) $$Foam\;stability\left(\%\right)=\frac{volume\;after\;30\;min}{volume\;after\;homogenization}\times 100$$

### 2.6. Physicochemical Characteristics of TKP-HD Foam-Mat Powder

#### Preparation of TKP-HD Foam-Mat Powder

The TKP-HD solution was mixed with hydroxypropyl methylcellulose (HPMC) at concentrations of 1.0% and 1.5% (*w*/*w*, by weight of TKP-HD solution) using a food processor (KM020, Kenwood, Havant, UK) at high speed for 3 min. The foam was spread evenly onto trays to a thickness of 1 cm and dried in a tray dryer at 60, 70, and 80 °C until the water activity was less than 0.3. The dried foam mats were then ground, packaged in aluminum zip-lock bags, and stored in a desiccator before analysis.

### 2.7. Determination of TKP-HD Foam-Mat Powder

#### 2.7.1. Chemical Composition

The samples were analyzed for moisture, protein, fat, and ash and carbohydrates were determined using AOAC methods [31].

#### 2.7.2. Water Activity

The water activity of the samples was measured using a water activity meter (AquaLab Series 3 TE, METER Group, Pullman, WA, USA).

#### 2.7.3. Antioxidants Activities

##### DPPH Free Radical Scavenging Assay

The DPPH radical scavenging activity of TKP-HD foam-mat powder was measured according to the method described by [1]. A 100 µM DPPH solution was dissolved in ethanol. A sample solution (1 mL) was mixed with the DPPH solution (2 mL), incubated in the dark for 1 h at room temperature, and the absorbance was measured at 515 nm. Antioxidant activity was expressed as µM Trolox equivalent/g sample using a Trolox calibration curve (0–100 µM).

##### Ferric Reducing Antioxidant Power (FRAP) Assay

The ferric reducing antioxidant power (FRAP) assay was determined as previously reported [1]. The FRAP reagent was prepared by mixing acetate buffer (pH 3.6), TPTZ solution (10 mM in 40 mM HCl), and FeCl_3_ (20 mM). A sample solution (0.5 mL) was reacted with FRAP reagent (2.5 mL) for 30 min in the dark, and the absorbance was measured at 593 nm. Antioxidant activity was expressed as µM Trolox equivalent/g sample.

#### 2.7.4. Solubility and Water-Binding Capacity

The solubility and water-binding capacity of the samples were evaluated following previously reported methods [33,34]. Briefly, samples (0.2 g) were dissolved in distilled water (40 mL), vortex-mixed for 1 min, and centrifuged at 4000× *g* for 15 min. Solubility and water-binding capacity were calculated using Equation (1) described previously.

#### 2.7.5. Viscosity

Sample viscosity was measured following the method of [35], with slight modifications. Samples (0.2 g) were dissolved in distilled water (40 mL), and the viscosity was determined using a Brookfield rotational viscometer (RVD-II+, Brookfield Engineering Laboratories Inc., Middleboro, MA, USA) equipped with a UL adapter and ULA spindle at 25 °C and 50 rpm, using a sample volume of 16 mL.

#### 2.7.6. Microstructure by Scanning Electron Microscopy

The samples were sputter-coated with gold before microstructural analysis using a field-emission scanning electron microscope (FE-SEM; JSM-7800F, JEOL Ltd., Tokyo, Japan) at an accelerating voltage of 15 kV and 500× magnification.

### 2.8. Statistical Analysis

All the experiments were performed following a completely randomized design (CRD), with results reported as mean ± standard deviation of three replicates. Statistical differences among treatments were evaluated by one-way analysis of variance (ANOVA), with Duncan’s multiple range test used at a significance level of *p* ≤ 0.05.

## 3. Results and Discussion

### 3.1. Functional Properties of TKP-HD

#### 3.1.1. Color

The color of food products is affected by ingredient composition, processing parameters, and storage conditions [36]. The color values of TKP-HD, EW, and SPI are presented in Table 1. The three proteins showed significant differences in L*, a*, and b* values. EW exhibited the highest lightness (L* = 88.07), whereas TKP-HD showed a lower L* value (78.53), and SPI presented the lowest L* value (76.59), indicating the darkest appearance among the samples. The a* value of EW was negative (−0.84), whereas positive a* values were observed for TKP-HD (1.31) and SPI (1.53 ± 0.03), indicating a shift toward redness. SPI exhibited the greatest yellowness (21.57), followed by EW, while TKP-HD showed the lowest b* value (13.21).

#### 3.1.2. Chemical Composition

The chemical compositions of TKP-HD, EW, and SPI are shown in Table 2. TKP-HD exhibited the lowest moisture content (4.18%), while the moisture content of EW (7.52%) was not significantly different from SPI (8.59%). SPI showed a significantly higher protein content (89.06%) than EW (81.83%) and TKP-HD (55.54%). Conversely, TKP-HD contained the highest fat content (3.44%) compared with both reference proteins, suggesting compositional differences related to protein source. This observation concurred with the findings of [1], who reported that tamarind kernel powder contained 20% protein and 5% fat. By contrast, EW and SPI exhibited protein contents of 80–90% and fat contents of 1–2% [37,38].

#### 3.1.3. Amino Acid Profiles

The amino acid profiles of TKP and TKP-HD (Table 3) show that TKP contained a higher proportion of hydrophobic amino acids (531.07 residues per 1000 residues) than hydrophilic amino acids (468.93 residues per 1000 residues). Glutamic acid was the predominant amino acid (156.47 residues per 1000 residues), followed by aspartic acid and glycine, accounting for 123.59 and 89.24 residues per 1000 residues, respectively. TKP-HD exhibited a lower proportion of hydrophobic amino acids (509.27 residues per 1000 residues) and a higher proportion of hydrophilic amino acids (490.73 residues per 1000 residues). This was attributed to the centrifugation step during protein extraction, where hydrophilic amino acids that preferentially dissolved in the supernatant were partially removed. Similarly to TKP, glutamic acid was the most abundant amino acid (154.08 residues per 1000 residues) in TKP-HD, followed by aspartic acid and glycine (114.26 and 98.69 residues per 1000 residues, respectively). The amino acid composition obtained in this study concurred with the findings of [39]. Cysteine was present at the lowest level in both TKP and TKP-HD at 4.71 and 8.23 residues per 1000 residues, respectively.

#### 3.1.4. Solubility and Water-Binding Capacity

**Figure 1:**
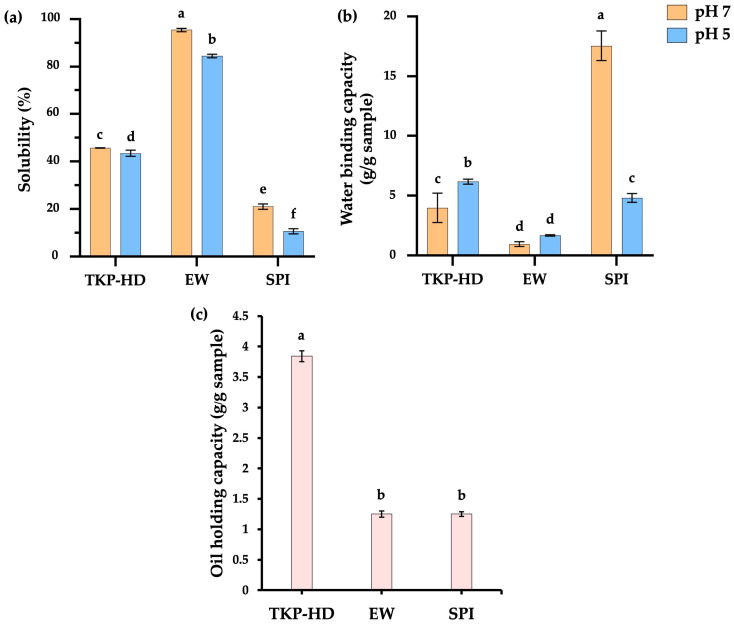
Solubility (**a**), water-binding capacity (**b**) and oil-holding capacity (**c**) of TKP-HD, EW, and SPI. Different letters indicate statistically significant differences (*p* ≤ 0.05).

Protein solubility is an essential functional property, describing the extent to which proteins dissolve in aqueous systems under different pH conditions (e.g., pH 5 and 7), and is a key indicator of protein quality and suitability for food applications [34]. As shown in Figure 1a, EW showed the greatest solubility at pH 7 (95.43%), primarily due to its high content of water-soluble globular proteins, such as ovalbumin, ovotransferrin, and ovomucin. The large amounts of polar and charged amino acids in EW proteins promoted interactions with water through hydrogen and ionic bonds [40,41]. TKP-HD exhibited 45.72% solubility, significantly lower than egg white protein (*p* ≤ 0.05). Previous studies reported that tamarind kernel powder contained 61.3% soluble protein, with only 19.6% water-soluble [42]. Enzymatic hydrolysis enhanced the solubility of TKP proteins by producing smaller peptide fractions [43]. Previous studies also found that enzymatic hydrolysis using alcalase improved protein solubility by decreasing surface hydrophobicity in peanut protein extracts [44]. SPI exhibited the lowest solubility due to the presence of hydrophobic proteins such as β-conglycinin (7S) and glycinin (11S) [45]. These proteins aggregate through hydrophobic interactions, leading to the formation of insoluble complexes with limited water solubility [46]. The low solubility of SPI was associated with the compact globular structure of its 7S and 11S fractions, characterized by low molecular flexibility and large molecular size [47,48]. At pH 5, protein solubility significantly decreased compared with results for pH 7 in all the samples, with TKP-HD, EW, and SPI giving solubilities of 43.49%, 84.46%, and 10.61%, respectively. This decrease in solubility was attributed to protein aggregation or precipitation near the isoelectric point (pI), with the pI values of EW and SPI 4.8 and 4.6, respectively [46,49]. At pH 5, the net charge of protein molecules decreased toward neutrality, reducing electrostatic repulsion and promoting molecular aggregation and precipitation, resulting in a decrease in solubility [50]. Similarly, Ferreira Machado et al. [40] suggested that reducing the pH of a solution resulted in a decrease in the solubility of EW, similar to SPI [51].

Water-binding capacity refers to the ability of a food matrix to absorb water and is directly linked to its ability to retain water within the matrix [52]. At pH 7, the water-binding capacity ranged from 0.95 to 17.54 (Figure 1b). EW exhibited the lowest value (0.95), which was not significantly different from that observed at pH 5 (*p* > 0.05) and was consistent with its solubility behavior (Figure 1a). Due to its high solubility, EW formed a limited amount of water-binding protein precipitate after centrifugation. TKP-HD exhibited increased water-binding capacity at pH 5, which was attributed to the closer proximity of protein molecules as the pH approached the isoelectric point (pI), promoting protein aggregation and subsequent water retention after centrifugation. Water interacts with proteins through several mechanisms, whereby water molecules surround protein structures and are retained by hydrogen bonding, as well as interactions with hydrophilic groups. Protein water binding primarily involves carboxyl, hydroxyl, carbonyl, and sulfhydryl polar hydrophilic groups [53]. At pH 7, SPI exhibited the maximum water-binding capacity (17.54), due to the formation of insoluble protein precipitates after centrifugation, which bound water via hydrogen bonding at the protein surface. At pH 5, the water-binding capacity decreased to 4.83 as the pH approached the isoelectric point (pI = 4.6) [46], resulting in the formation of hexamers of the β-conglycinin protein, while the decreased net charge of the protein molecules led to increased protein–protein interactions, causing protein molecules to move closer together and lose their water-binding capacity [54].

#### 3.1.5. Oil-Holding Capacity

Oil-holding capacity (OHC) is defined as the ability to absorb and bind oil within the protein matrix. The OHC values of TKP-HD, EW, and SPI are shown in Figure 1c. TKP-HD exhibited the highest OHC (3.84 g oil/g sample). The unfolding of protein structures induced by high-pressure processing exposes functional groups capable of interacting with hydrocarbon chains of oil via hydrophobic interactions, electrostatic interactions, and hydrogen bonding [34]. The OHC values of EW and SPI were not significantly different (*p* > 0.05). TKP-HD contained a higher proportion of hydrophobic amino acids than TKP (Table 3), which contributed to its enhanced oil-holding capacity.

#### 3.1.6. Foam Capacity and Foam Stability

**Figure 2:**
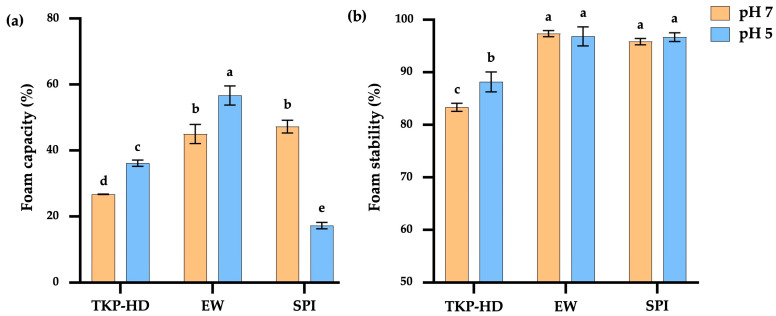
Foam capacity (**a**) and foam stability (**b**) of TKP-HD, EW, and SPI. Different letters indicate statistically significant differences (*p* ≤ 0.05).

Foam capacity is a key functional property of proteins that plays an important role in determining the texture and mouthfeel of aerated food products, such as meringues, cakes, and whipping creams [34]. The functionality of proteins in foam systems involves reducing surface tension, increasing the viscosity and viscoelasticity of the liquid phase, and forming a strong interfacial film at the air–liquid interface [55] (pp. 260–309). As illustrated in Figure 2a, at pH 7.0, EW and SPI exhibited foam capacities of 45.0% and 47.22%, respectively, with no significant difference (*p* > 0.05). Conversely, TKP-HD showed the lowest foam capacity (26.67%), due to its lower protein content (Table 2). According to [56], increasing protein concentration enhances foam capacity by promoting the formation of a thicker interfacial film that is smoother, denser, and more stable than films formed at lower protein concentrations, thereby enhancing protein adsorption during mixing and improving the efficiency of air bubble encapsulation. EW can form stable foams due to its amphiphilic behavior [57]. EW proteins readily disperse in both the liquid and air phases, where air bubbles are enveloped by a continuous thin protein film and separated by lamellae, contributing to foam stability [58]. At pH 5, EW and TKP-HD exhibited increased foam capacities of 56.67% and 26.11%, respectively. This behavior was attributed to enhanced protein–protein interactions near the isoelectric point (pI), leading to the formation of a thicker interfacial protein film with near electrical neutrality at the water-air bubble interface and reduced electrostatic repulsion between protein molecules [59]. SPI exhibited a lower foam capacity (17.22%), with protein aggregation at pH 5 and decreased solubility at lower pH values (Figure 1a). Reduced protein solubility limits interfacial adsorption, thereby decreasing foam capacity [60]. Differences in foam capacity among the proteins were associated with variations in amino acid composition and sequence, molecular size, protein conformation, surface electrical properties, net charge, and hydrophobicity.

EW and SPI foams exhibited the highest foam stability (97.36% and 95.85%, respectively) at pH 7, and these values were not significantly different at pH 5. This stability was attributed to the elastic protein film that encapsulates air bubbles, as both foam capacity and foam stability are determined by the ability of proteins to form elastic interfacial films capable of binding air bubbles and resisting mechanical stress during whipping and dehydration [56] (pp. 260–309). TKP-HD showed the lowest foam stability at pH 7, a behavior that was associated with its lower protein concentration compared with EW and SPI (Table 2). At high protein concentrations, proteins can form a thicker interfacial film, resulting in smoother, denser, and more stable foams. Proteins readily adsorb at the air–liquid interface, allowing air bubbles to be effectively encapsulated by a stable protein film. By contrast, TKP-HD showed increased foam stability at pH 5, attributed to the closer proximity of protein molecules and enhanced protein–protein interactions as the pH approached the isoelectric point of TKP (pI = 4–5) [9]. Protein hydrolysis results in the breakdown of proteins into peptides or smaller amino acids. These peptides readily adsorb at the air–water interface, but they often lack the ability to form a cohesive and elastic interfacial film, and protein aggregation occurring as the pH approaches the isoelectric point (pI) promotes the formation of a more elastic and stable interfacial film [61].

### 3.2. Physicochemical Characteristics of TKP-HD Foam-Mat Powder

#### 3.2.1. Chemical Composition and Water Activity

The chemical compositions of TKP-HD foam-mat powders at different drying temperatures and HPMC concentrations are shown in Table 4, including moisture, protein, fat, ash, and carbohydrate contents. Moisture content was not significantly influenced by HPMC concentration, whereas drying at 80 °C resulted in a significant reduction in moisture content (*p* ≤ 0.05). A high temperature difference between the TKP-HD foam and the drying air enhanced heat and mass transfer, leading to rapid moisture removal [62]. The protein content ranged from 35 to 38.75%. The sample containing 1% HPMC and dried at 70 °C exhibited the highest protein content, which was not significantly different from samples containing 1% HPMC dried at 60 and 80 °C (*p* > 0.05). By contrast, the addition of HPMC at 1.5% resulted in decreased protein and fat contents but significantly increased ash and carbohydrate contents due to the increased dry solids contributed by HPMC.

**Figure 4:**
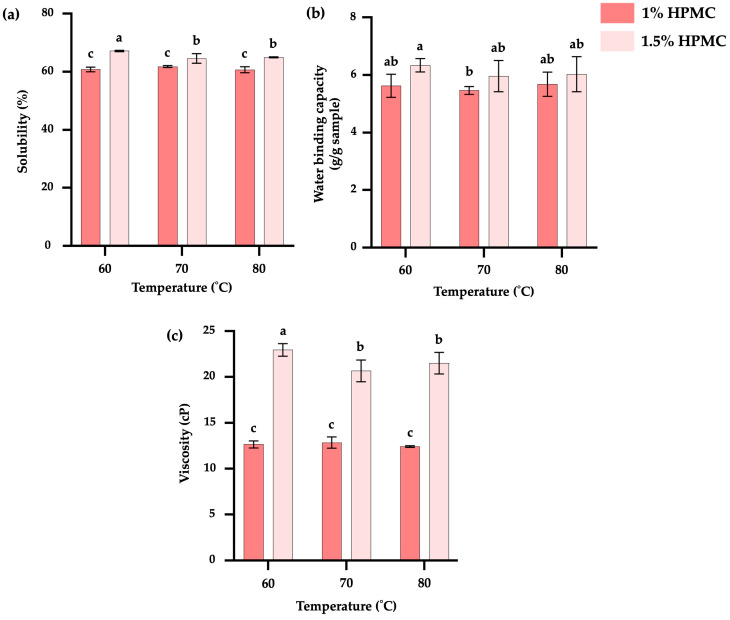
Solubility (**a**), water-binding capacity (**b**), and viscosity (**c**) of TKP-HD foam-mat powder at different drying temperatures and HPMC concentrations. Different letters indicate statistically significant differences (*p* ≤ 0.05).

Table 4 shows the water activity of TKP-HD foam-mat powder at different drying temperatures and HPMC concentrations. Samples containing 1.5% HPMC exhibited significantly lower water activity than those containing 1% HPMC across all drying temperatures (60, 70, and 80 °C) (*p* ≤ 0.05). At the same HPMC concentration, drying at 80 °C produced lower water activity than drying at 70 °C. The decrease in water activity was attributed to the loss of moisture content at higher drying temperatures, as the food structure became more porous, enhancing moisture removal. Protein unfolding induced by thermal denaturation reduced the water-holding capacity and released immobilized water within the structure, thereby further decreasing water activity [63]. HPMC improved the viscosity and elasticity of the foam matrix, contributing to foam stability. During drying, as moisture evaporated (Figure 4c), the increased viscosity stabilized the foam structure, resulting in a strengthened foam network with higher porosity, which facilitated greater moisture removal from the structure [64].

#### 3.2.2. Antioxidant Activities

##### DPPH Free Radical Scavenging Assay

**Figure 3:**
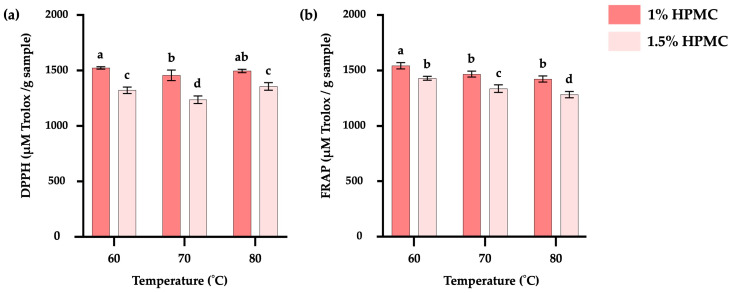
DPPH assay results (**a**) and FRAP assay results (**b**) of TKP-HD foam-mat powder at different drying temperatures and HPMC concentrations. Different letters indicate statistically significant differences (*p* ≤ 0.05).

Figure 3a illustrates the antioxidant activity of TKP-HD foam-mat powder at different drying temperatures and HPMC concentrations, evaluated using the DPPH free radical scavenging assay. Samples containing 1% HPMC and dried at 60 °C exhibited the highest antioxidant activity (1523.75 µM Trolox/g), which was not significantly different from samples dried at 80 °C at the same concentration (1496.02 µM Trolox/g) (*p* > 0.05). The antioxidant activity of the samples decreased as the drying temperature increased from 60 to 70 °C. This reduction may be associated with the formation of low-molecular-weight peptides and the presence of Maillard reaction products generated during the drying processing [65]. The addition of HPMC at 1.5% decreased antioxidant activity, attributed to the reduced protein concentration (Table 4). The antioxidant activity of TKP protein hydrolysate powder produced by papain hydrolysis was attributed to the production of peptides that donated protons and electrons during peptide bond cleavage. Proton donation involved in free radical stabilization may occur through amino acid side-chain residues or specific peptide conformations [66]. Therefore, the decrease in protein content resulted in reduced peptide and amino acid contents, thereby lowering the availability of hydrogen-donating groups capable of scavenging DPPH radicals. At an HPMC concentration of 1.5%, samples dried at 80 °C showed higher antioxidant activity than those dried at 70 °C. This effect was related to the shorter drying time associated with high-temperature foam-mat drying, which restricted the degradation of antioxidant compounds [67].

##### Ferric Reducing Antioxidant Power (FRAP) Assay

Figure 3b illustrates the antioxidant activity of TKP-HD foam-mat powder at different drying temperatures and HPMC concentrations, evaluated using the ferric reducing antioxidant power (FRAP) assay. The highest ferric reducing antioxidant power was observed in samples containing 1% HPMC and dried at 60 °C, consistent with the results of the DPPH assay. A reduction in antioxidant activity was observed with increasing drying temperature and HPMC concentration, following trends similar to those discussed for the DPPH results.

#### 3.2.3. Solubility

The solubility of TKP-HD foam-mat powder (Figure 4a) ranged from 60.67 to 67.14%. The sample dried at 60 °C with 1.5% HPMC exhibited the highest solubility (67.14%). Samples containing 1% HPMC showed lower solubility than those containing 1.5% HPMC at the same drying temperature. The solubility of the foam-mat powder was attributed to the porous structure formed during drying. The addition of HPMC increased mixture viscosity (Figure 4c), which stabilized the foam structure and preserved it throughout the drying process. As a result, the collapse and coalescence of neighboring air bubbles were reduced, resulting in a more uniform pore structure and pore size distribution [63,68]. At lower drying temperatures, protein denaturation was limited, allowing hydrophobic regions to remain buried within the structure; consequently, the sample containing 1.5% HPMC and dried at 60 °C exhibited the highest solubility.

#### 3.2.4. Water-Binding Capacity

Water-binding capacity determines the amount of water absorbed by dried powders and is closely associated with rehydration ability. No significant differences (*p* > 0.05) in water-binding capacity were observed among the samples, except for the foam-mat powder containing 1.5% HPMC and dried at 60 °C, which had the highest value in WBC (Figure 4b). Water-binding capacity is associated with the multilayered arrangement of water molecules surrounding proteins, which promotes water–water interactions and enhances the solubility of protein particles. Protein water binding occurs primarily through hydrogen bonding involving hydrophilic groups. This capacity depends on several factors, including the availability of binding sites on the particle surface, particle size, and the number of hydrophilic groups present. Native proteins generally exhibit higher water-binding capacity than denatured or modified proteins [56] (pp. 76–133). At the lower drying temperature (60 °C), protein denaturation was reduced, resulting in the highest water-binding capacity. Azizpour et al. [63] suggested that a decrease in water-binding capacity at higher drying temperatures may be attributed to protein denaturation, which causes hydrophobic groups to migrate to the protein surface and reduces hydrogen bonding with water. When extensive denaturation occurs, accompanied by protein agglomeration and particle aggregation, water absorption decreases due to the reduced interfacial surface area available for protein–water interactions.

#### 3.2.5. Viscosity

Viscosity is an important parameter in liquid foods, as it indicates changes during processing and quality control and is closely related to consumer sensory perception [69]. In Figure 4c, the sample dried at 80 °C with 1% HPMC exhibited the lowest viscosity (12.42 cP), which was not significantly different from samples dried at 60 and 70 °C. By contrast, increasing the HPMC concentration to 1.5% resulted in significantly higher viscosity for powders dried at all temperatures (*p* ≤ 0.05). The results indicated that foaming agent concentration influenced the viscosity after reconstitution and also during the whipping process. Improved foam stability at appropriate viscosity levels helps prevent the coalescence of air bubble walls, thereby enhancing foam stability [70]. This behavior concurred with the higher solubility observed in the samples (Figure 4a). When proteins are dissolved in a solvent, they can interact with solvent molecules through multiple mechanisms, including hydrogen bonding, electrostatic interactions, and hydrophobic interactions. These interactions may induce protein unfolding or conformational changes from the native structure, thereby increasing solution viscosity through enhanced interactions between hydrophilic regions and water molecules [71]. At lower drying temperatures (60 °C), protein denaturation was reduced, allowing hydrophobic regions to remain buried within the protein molecules.

#### 3.2.6. Microstructure by SEM

**Figure 5:**
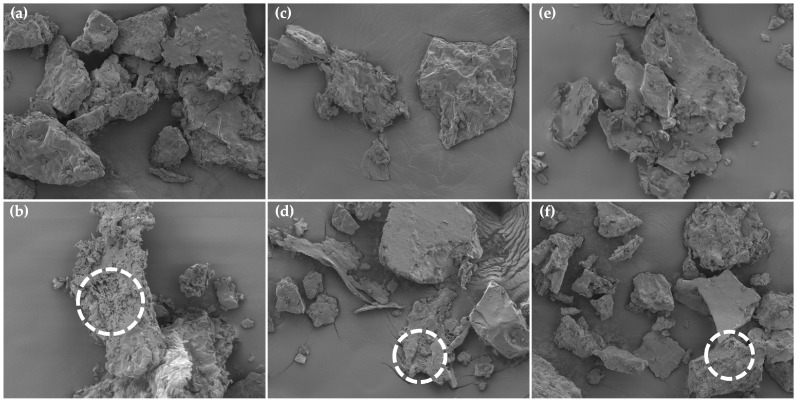
SEM images of TKP-HD foam-mat powder at different drying temperatures and HPMC concentrations at 500× magnification. (**a**) 60 °C/1% HPMC; (**b**) 60 °C/1.5% HPMC; (**c**) 70 °C/1% HPMC; (**d**) 70 °C/1.5% HPMC; (**e**) 80 °C/1% HPMC; (**f**) 80 °C/1.5% HPMC.

The microstructure of TKP-HD foam-mat powder is shown in Figure 5. The addition of 1.5% HPMC produced a more porous structure with a higher number of entrapped air bubbles (Figure 5, circled area). When compared with the addition of 1% HPMC. The hydrolysate powder containing 1.5% HPMC and dried at 60 °C exhibited a highly porous protein structure with numerous pores (Figure 5, circled area). This structure reduced the collapse and aggregation of air bubbles at lower drying temperatures. Our results indicated that a low concentration of foaming agent produced a thinner interfacial film that was more prone to collapse [72]. By contrast, a higher HPMC concentration increased foam viscosity, resulting in improved structural stability and facilitating moisture release from the foam structure before collapse [71]. The solubility of TKP-HD foam-mat powder (Figure 4a) was consistent with the microstructural observations obtained by SEM. As mentioned previously, higher powder porosity increased the available surface area for interactions between proteins and water.

## 4. Conclusions

High-pressure assisted protein hydrolysate from tamarind kernel powder (TKP-HD) exhibited distinctive functional and physicochemical properties in its foam-mat powder form. TKP-HD consisted of more non-polar than polar amino acids, with higher solubility than SPI but lower solubility than EW. The WBC of TKP-HD increased at pH 5. The oil-binding capacity of TKP-HD was greater than the other proteins. TKP-HD had a higher foaming capacity than SPI at pH 5 but exhibited lower foam stability than the other two proteins at pH 5 and 7. The physicochemical characteristics of TKP-HD with foam-mat drying indicated that higher drying temperatures decreased antioxidant activity by the FRAP assays compared to 60 °C. Furthermore, drying at a lower temperature (60 °C) increased the antioxidant activity by the DPPH assays. The addition of 1.5% HPMC decreased antioxidant activities, protein content, and water activity, contrasting with the higher solubility and more porous structure of the dried powder. From this study, the optimal condition for foam-mat TKP-HD drying was determined as HPMC 1.5% with drying at 60 °C.

From an industrial perspective, applying HPP-assisted enzymatic hydrolysis and foam-mat drying offers both promising opportunities and practical challenges. While these processes may require higher capital investment and additional processing steps, the observed improvements in functional performance suggest that further evaluation is worthwhile, particularly for value-added applications. Practical aspects such as energy consumption, processing time, and the feasibility of scale-up should be carefully examined in future pilot-scale studies.

## Figures and Tables

**Table 1:** Color value (L* a* b*) of TKP-HD, EW, and SPI.

Sample	Color Value		
	L*	a*	b*
TKP-HD	78.53 ^b^ ± 1.34	1.31 ^b^ ± 0.53	13.21 ^c^ ± 0.12
EW	88.07 ^a^ ± 0.11	−0.84 ^c^ ± 0.23	14.36 ^b^ ± 0.23
SPI	76.59 ^c^ ± 0.36	1.53 ^a^ ± 0.03	21.57 ^a^ ± 0.42

Values are expressed as mean ± SD of three replications. Values in each column with different lowercase superscripts (a–c) are significantly (*p* ≤ 0.05) different.

**Table 2:** Chemical compositions of TKP-HD, EW, and SPI.

Sample	Moisture Content (%)	Protein Content (%)	Fat Content (%)
TKP-HD	4.18 ^b^ ± 0.23	55.54 ^c^ ± 0.72	3.44 ^a^ ± 0.30
EW	7.52 ^a^ ± 0.56	81.83 ^b^ ± 3.46	1.03 ^c^ ± 0.09
SPI	8.59 ^a^ ± 1.01	89.06 ^a^ ± 3.29	1.91 ^b^ ± 0.12

Values are expressed as mean ± SD of three replications. Values in each column with different lowercase superscripts (a–c) are significantly (*p* ≤ 0.05) different.

**Table 3:** Amino acid composition of TKP and TKP-HD.

Amino Acid	TKP	TKP-HD
	(Residues/1000 Residues)	
Aspartic acid	123.59	114.26
Threonine	37.75	38.34
Serine	76.26	75.03
Glutamic acid	156.47	154.08
Lysine	62.39	62.29
Histidine	17.59	17.16
Arginine	57.01	48.13
Glycine	89.24	98.69
Alanine	66.55	65.22
Valine	44.46	47.37
Cysteine	4.71	8.23
Methionine	11.84	12.02
Isoleucine	44.07	45.16
Leucine	83.04	83.4
Tyrosine	33.95	38.07
Phenylalanine	39.30	40.42
Proline	51.77	52.15
Total	1000.00	1000.00
Hydrophobic amino acids	531.07	509.27
Hydrophilic amino acids	468.93	490.73

**Table 4:** Chemical composition and water activity of TKP-HD foam-mat powder at different drying temperatures and HPMC concentrations.

Temperature (°C)	HPMC Concentration (%)	Chemical Composition (%Wet Basis)					Water Activity
		Moisture	Protein	Fat	Ash	Carbohydrate	
60	1	3.73 ^a^ ± 0.14	38.35 ^a^ ± 1.03	3.16 ^a^ ± 0.09	3.49 ^a^ ± 0.18	51.27 ^c^ ± 1.25	0.30 ^a^ ± 0.00
	1.5	3.45 ^ab^ ± 0.56	35.00 ^c^ ± 0.19	2.17 ^b^ ± 0.08	5.25 ^b^ ± 0.14	54.14 ^a^ ± 0.48	0.27 ^c^ ± 0.00
70	1	3.58 ^a^ ± 0.26	38.75 ^a^ ± 0.23	3.12 ^a^ ± 0.07	3.52 ^a^ ± 0.30	51.03 ^c^ ± 0.31	0.30 ^a^ ± 0.00
	1.5	3.48 ^ab^ ± 0.31	36.13 ^b^ ± 0.42	2.07 ^b^ ± 0.07	5.11 ^b^ ± 0.59	53.22 ^ab^ ± 1.07	0.29 ^b^ ± 0.01
80	1	3.00 ^bc^ ± 0.27	38.38 ^a^ ± 0.04	3.17 ^a^ ± 0.01	3.49 ^a^ ± 0.04	51.94 ^bc^ ± 0.32	0.29 ^b^ ± 0.00
	1.5	2.78 ^c^ ± 0.32	35.33 ^bc^ ± 0.42	2.16 ^b^ ± 0.06	5.38 ^b^ ± 0.18	54.36 ^a^ ± 0.18	0.27 ^c^ ± 0.01

Values are expressed as mean ± SD of three replications. Values in each column with different lowercase superscripts (a–c) are significantly (*p* ≤ 0.05) different.

## Data Availability

The original contributions presented in the study are included in the article. Further inquiries can be directed to the corresponding author.

## References

[1] Sricheevachart K., Piyachomkawn K., Sompongse W. (2023). Effects of high-pressure processing on techno-functional properties of tamarind (*Tamarindus indica* L.) kernel powder. Int. J. Food Sci. Technol..

[2] Nagar C.K., Dash S.K., Rayaguru K. (2022). Tamarind seed: Composition, applications, and value addition: A comprehensive review. J. Food Process. Preserv..

[3] Tangariya P., Srivastava S. (2022). Comparison of functional properties of tamarind kernel powder with whole wheat flour, lentil powder and evaluation of sensory and nutritional quality of tamarind kernel powder incorporated cookies. Pharma Innov. J..

[4] Vutthidech T., Sompongse W. The effects of high pressure-assisted enzymatic hydrolysis on degree of hydrolysis and antioxidant properties of tamarind kernel protein. Proceedings of the 25th Food Innovation Asia Conference 2023 (FIAC 2023), BITEC.

[5] Geethalaxmi M., Sunil C.K., Venkatachalapathy N. (2024). Tamarind seed polysaccharides, proteins, and mucilage: Extraction, modification of properties, and their application in food. Sustain. Food Technol..

[6] Shalini P., Siddalinga Murthy K.R. (2018). Isolation of tamarind seed proteins, polysaccharides, and cellulase from the seeds of *Tamarindus indica*. Pharma Innov. J..

[7] Bagul M.B., Sonawane S.K., Arya S.S. (2018). Bioactive characteristics and optimization of tamarind seed protein hydrolysate for antioxidant-rich food formulations. 3 Biotech.

[8] Smith A.K., Circle C.J. (1939). Soybean protein precipitation from water and alkaline dispersions by acids and by electrodialysis. Ind. Eng. Chem..

[9] Biswas B., Sit N. (2020). Effect of ultrasonication on functional properties of tamarind seed protein isolates. J. Food Sci. Technol..

[10] Fadimu G.J., Le T.T., Gill H., Farahnaky A., Olatunde O.O., Truong T. (2022). Enhancing the Biological Activities of Food Protein-Derived Peptides Using Non-Thermal Technologies: A Review. Foods.

[11] Gouseti O., Larsen M.E., Amin A., Bakalis S., Petersen I.L., Lametsch R., Jensen P.E. (2023). Applications of enzyme technology to enhance transition to plant proteins: A review. Foods.

[12] Vogelsang-O’Dwyer M., Sahin A.W., Zannini E., Arendt E.K. (2022). Enzymatic hydrolysis of pulse proteins as a tool to improve techno-functional properties. Foods.

[13] Verma V., Singhal G., Joshi S., Choudhary M., Srivastava N., Mir S.A., Manickavasagan A., Shah M.A. (2022). Plant extracts as enzymes. Plant Extracts: Applications in the Food Industry.

[14] Landim A.P.M., Tiburski J.H., Mellinger C.G., Juliano P., Rosenthal A. (2023). Potential application of high hydrostatic pressure on the production of hydrolyzed proteins with antioxidant and antihypertensive properties and low allergenicity: A review. Foods.

[15] Zhao F., Liu X., Lian M., Yang Y., Li C., Xu H., Wang W. (2023). Effects of high hydrostatic pressure on physicochemical and functional properties of soybean protein isolate. Food Sci. Technol..

[16] Lullien-Pellerin V., Balny C. (2002). High-pressure as a tool to study some proteins’ properties: Conformational modification, activity and oligomeric dissociation. Innov. Food Sci. Emerg. Technol..

[17] Goyal A., Sharma V., Upadhyay N., Sihag M., Kaushik R. (2013). High pressure processing and its impact on milk proteins: A review. J. Dairy Sci. Technol..

[18] Yang J., Powers J.R., Balasubramaniam V.M., Barbosa-Cánovas G.V., Lelieveld H.L.M. (2016). Effects of high pressure on food proteins. High Pressure Processing of Food: Principles, Technology and Applications.

[19] Landim A.P.M., Chávez D.W.H., Rosa J.S.D., Mellinger-Silva C., Rosenthal A. (2021). Effect of high hydrostatic pressure on the antioxidant capacity and peptic hydrolysis of whey proteins. Cienc. Rural.

[20] Franck M., Perreault V., Suwal S., Marciniak A., Bazinet L., Doyen A. (2019). High hydrostatic pressure-assisted enzymatic hydrolysis improved protein digestion of flaxseed protein isolate and generation of peptides with antioxidant activity. Food Res. Int..

[21] de Carvalho Oliveira L., Martinez-Villaluenga C., Frias J., Cartea M.E., Francisco M., Cristianini M., Penas E. (2024). High pressure-assisted enzymatic hydrolysis potentiates the production of quinoa protein hydrolysates with antioxidant and ACE-inhibitory activities. Food Chem..

[22] Ahmed J., Mulla M., Al-Ruwaih N., Arfat Y.A. (2019). Effect of high-pressure treatment prior to enzymatic hydrolysis on rheological, thermal, and antioxidant properties of lentil protein isolate. Legume Sci..

[23] Bandyopadhyay M., Guha S., Naldrett M.J., Alvarez S., Majumder K. (2021). Evaluating the effect of high-pressure processing in contrast to boiling on the antioxidant activity from alcalase hydrolysate of Great Northern beans (*Phaseolus vulgaris*). J. Food Biochem..

[24] Kalambe S., Guhe S. (2026). Foam mat drying of perishable products: A critical review of process parameters, product quality, and sustainable prospects. Sustain. Food Technol..

[25] Sangamithra A., Venkatachalam S., John S.G., Kuppuswamy K. (2015). Foam mat drying of food materials: A review. J. Food Process. Preserv..

[26] Hardy Z., Jideani V.A. (2017). Foam-mat drying technology: A review. Crit. Rev. Food Sci. Nutr..

[27] Kandasamy P., Varadharaju N., Kalemullah S., Maladhi D., Moorthy I.G., Sivasubramanian V. (2014). Optimization of process parameters for foam-mat drying of papaya pulp. J. Food Sci. Technol..

[28] Buljat A.M., Jurina T., Jurinjak Tušek A., Valinger D., Gajdoš Kljusurić J., Benković M. (2019). Applicability of foam mat drying process for production of instant cocoa powder enriched with lavender extract. Food Technol. Biotechnol..

[29] Sukkhown P., Jangchud K., Lorjaroenphon Y., Pirak T. (2018). Flavored-functional protein hydrolysates from enzymatic hydrolysis of dried squid by-products: Effect of drying method. Food Hydrocoll..

[30] Sritongtae B., Karami Z., Morgan M.R., Duangmal K. (2022). Fractionation of foam-mat dried rice bean hydrolysates using membrane filtration and solid phase extraction: Peptide- and phenolic-based fractions with bioactive potential. Food Res. Int..

[31] AOAC (2000). Official Methods of Analysis.

[32] Sompongse W., Morioka K., Itoh Y. (2003). Comparison of amino acid composition among various surimis and washed meats. Res. Rep. Kochi Univ. Agric..

[33] Sosulski F.W. (1962). The centrifuge method for determining flour absorptivity in hard red spring wheats. Cereal Chem..

[34] Eckert E., Han J., Swallow K., Tian Z., Jarpa-Parra M., Chen L. (2019). Effects of enzymatic hydrolysis and ultrafiltration on physicochemical and functional properties of faba bean protein. Cereal Chem..

[35] Charunuch C., Tangkanakul P., Limsangouan N., Sonted V. (2008). Effects of extrusion conditions on the physical and functional properties of instant cereal beverage powders admixed with mulberry (*Morus alba* L.) leaves. Food Sci. Technol. Res..

[36] Pathare P.B., Opara U.L., Al-Said F.A.J. (2013). Colour measurement and analysis in fresh and processed foods: A review. Food Bioprocess Technol..

[37] Yu Y., Guan S., Li X., Sun B., Lin S., Gao F. (2023). Physicochemical and functional properties of egg white peptide powders under different storage conditions. J. Food Sci. Technol..

[38] L’hocine L., Boye J.I., Arcand Y. (2006). Composition and functional properties of soy protein isolates prepared using alternative defatting and extraction procedures. J. Food Sci..

[39] Kumar C.S., Bhattacharya S. (2008). Tamarind seed: Properties, processing and utilization. Crit. Rev. Food Sci. Nutr..

[40] Ferreira Machado F., Coimbra J.S.R., Garcia Rojas E.E., Minim L.A., Oliveira F.C., Sousa R.d.C.S. (2007). Solubility and density of egg white proteins: Effect of pH and saline concentration. LWT Food Sci. Technol..

[41] Chang C., Lahti T., Tanaka T., Nickerson M.T. (2018). Egg proteins: Fractionation, bioactive peptides and allergenicity. J. Sci. Food Agric..

[42] Joseph J., Kanchalochana S.N., Rajalakshmi G., Hari V., Durai R.D. (2012). Tamarind seed polysaccharide: A promising natural excipient for pharmaceuticals. Int. J. Green Pharm..

[43] Tavano O.L. (2013). Protein hydrolysis using proteases: An important tool for food biotechnology. J. Mol. Catal. B Enzym..

[44] Zhao G., Liu Y., Zhao M., Ren J., Yang B. (2011). Enzymatic hydrolysis and their effects on conformational and functional properties of peanut protein isolate. Food Chem..

[45] Wang T., Yi K., Li Y., Wang H., Fan Z., Jin H., Xu J. (2023). Esterified soy proteins with enhanced antibacterial properties for the stabilization of nano-emulsions under acidic conditions. Molecules.

[46] O’Flynn T.D., Hogan S.A., Daly D.F., O’Mahony J.A., McCarthy N.A. (2021). Rheological and solubility properties of soy protein isolate. Molecules.

[47] Lee K.H., Ryu H.S., Rhee K.C. (2003). Protein solubility characteristics of commercial soy protein products. J. Am. Oil Chem. Soc..

[48] Lee H., Yildiz G., Dos Santos L.C., Jiang S., Andrade J.E., Engeseth N.J., Feng H. (2016). Soy protein nano-aggregates with improved functional properties prepared by sequential pH treatment and ultrasonication. Food Hydrocoll..

[49] Abdel-Shafi S., Osman A., Enan G., El-Nemer M., Sitohy M. (2016). Antibacterial activity of methylated egg white proteins against pathogenic G^+^ and G^−^ bacteria matching antibiotics. SpringerPlus.

[50] Pelegrine D.H.G., Gasparetto C.A. (2005). Whey proteins solubility as a function of temperature and pH. LWT Food Sci. Technol..

[51] Jiang J., Xiong Y.L., Chen J. (2010). pH shifting alters solubility characteristics and thermal stability of soy protein isolate and its globulin fractions in different pH, salt concentration, and temperature conditions. J. Agric. Food Chem..

[52] Traynham T.L., Myers D.J., Carriquiry A.L., Johnson L.A. (2007). Evaluation of water-holding capacity for wheat–soy flour blends. J. Am. Oil Chem. Soc..

[53] Kinsella J.E., Morr C.V. (1984). Milk proteins: Physicochemical and functional properties. Crit. Rev. Food Sci. Nutr..

[54] Ochiai-Yanagi S., Miyauchi H., Saio K., Watanabe T. (1978). Modified soybean protein with high water-holding capacity. Cereal Chem..

[55] Zayas J.F. (1997). Functionality of Proteins in Food.

[56] Wang Y.H., Lin Y., Yang X.Q. (2019). Foaming properties and air–water interfacial behavior of corn protein hydrolyzate–tannic acid complexes. J. Food Sci. Technol..

[57] Segura-Campos M., Pérez-Hernández R., Chel-Guerrero L., Castellanos-Ruelas A., Gallegos-Tintoré S., Betancur-Ancona D. (2013). Physicochemical and functional properties of dehydrated Japanese quail (*Coturnix japonica*) egg white. Food Nutr. Sci..

[58] Du L., Prokop A., Tanner R.D. (2002). Effect of denaturation by preheating on the foam fractionation behavior of ovalbumin. J. Colloid Interface Sci..

[59] Kuropatwa M., Tolkach A., Kulozik U. (2009). Impact of pH on the interactions between whey and egg white proteins as assessed by the foamability of their mixtures. Food Hydrocoll..

[60] Sorgentini D.A., Wagner J.R. (2002). Comparative study of foaming properties of whey and isolate soybean proteins. Food Res. Int..

[61] Kinsella J.E. (1981). Functional properties of proteins: Possible relationships between structure and function in foams. Food Chem..

[62] Hamzeh S., Motamedzadegan A., Shahidi S.-A., Ahmadi M., Regenstein J.M. (2019). Effects of drying condition on physico-chemical properties of foam-mat dried shrimp powder. J. Aquat. Food Prod. Technol..

[63] Azizpour M., Mohebbi M., Khodaparast M.H.H. (2016). Effects of foam-mat drying temperature on physico-chemical and microstructural properties of shrimp powder. Innov. Food Sci. Emerg. Technol..

[64] Arboleya J., Wilde P. (2005). Competitive adsorption of proteins with methylcellulose and hydroxypropyl methylcellulose. Food Hydrocoll..

[65] Lee K.G., Shibamoto T. (2002). Toxicology and antioxidant activities of non-enzymatic browning reaction products: Review. Food Rev. Int..

[66] Wang L.L., Xiong Y.L. (2005). Inhibition of lipid oxidation in cooked beef patties by hydrolyzed potato protein is related to its reducing and radical scavenging ability. J. Agric. Food Chem..

[67] Sritongtae B., Morgan M., Duangmal K. (2017). Drying kinetics, physico-chemical properties, antioxidant activity and phenolic composition of foam-mat dried germinated rice bean (*Vigna umbellata*) hydrolysate. Int. J. Food Sci. Technol..

[68] Falade K.O., Olugbuyi A.O. (2010). Effects of maturity and drying method on the physico-chemical and reconstitution properties of plantain flour. Int. J. Food Sci. Technol..

[69] Padmanabhan M. (1995). Measurement of extensional viscosity of viscoelastic liquid foods. J. Food Eng..

[70] Karim A.A., Wai C.C. (1999). Foam-mat drying of starfruit (*Averrhoa carambola* L.) purée. Stability and air drying characteristics. Food Chem..

[71] Krasaekoopt W., Bhatia S. (2012). Production of yogurt powder using foam-mat drying. AU J. Technol..

[72] Sullca Grimaldez L., Martínez K.D. (2021). Concentration trend study on foaming properties for native soy protein isolate treated by ultrasound and heating. J. Food Sci. Technol..

